# The Indian Hemp Drug Commission

**Published:** 1895-09

**Authors:** 


### The Indian Hemp Drug Commission.

The report of the opium commission has effectually dispelled the cloud of delusion and prejudice with which the opium question, in its relation, both to India and China, has been for so many years invested by well-meaning but strangely-misinformed philanthropists. Opium must no longer be branded as a curse to man, destroying his body and ruining his soul, but thankfully accepted as a means of rendering less rugged and irksome the weary path of millions of men in their toilsome journey from birth to death. Accordingly the commission has not seen its way to recommened any modification of the existing regulations and usages relating to the cultivation of the poppy, the manufacture of opium, or its distribution by commerce.

One of the arguments used by those who do not believe in making people virtuous by legislation, or depriving them of articles which they may consider good or pleasant is, that man universally uses some neurotic, and that if we deprive him of that which is comparatively innocuous, we drive him to the consumption of some other nervine stimulant or sedative which is more harmful, physically and morally, to hemp and alcohol in the case of India, to alcohol in the case of China.

Hemp has been represented as a specially noxious substitute or alternative for opium in India. The use of haschish has been credited with terrible effects, violence, debauchery, insanity and crime. The jails and mad-houses of India have been said to be largely filled with its victims, and rape and murder alleged to be frequent and due to excess in smoking of ganja and drinking of bhang.

The report of the Hemp Drugs Commission, which has recently been published in India, has clearly demonstrated that this view of the effects of the consumption of hemp is grossly exaggerated, and that the evils commonly attributed to its use do not exist, and that the baneful effects caused by its abuse are very rare. It has been shown that in some parts of India the use of hemp in moderation is exceedingly common, as many as 1 in 200 of the inhabitants of Bengal consuming it much as we in this country consume tobacco, tea or coffee, or the inhabitants of France and Italy consume their cider or light wine, as a mild stimulant and harmless luxury. Not more than 1 in 4,000 inhabitants exceeds the bounds of strict moderation. "Moderate consumers," it is stated, "are not offensive as a rule, and, indeed, are not distinguishable from total abstainers,'' while as regards the few who use hemp drugs in excess ''there are no such marked ill-effects, physical, mental, or moral, attendant on their use as there were popularly believed to be before the present inquiry was made.'' ''To a million people in India,'' it is added, ''ganja affords a harmless pleasure, and in some cases even a beneficial stimulant.'' It is adapted to the constitution, occupations, and habits of Indian people, and the curious statement is made that the introduction into India of more severe industries, such as those connected with mills and mines, has compelled the workers to resort to the "fiercer stimulant of alcohol.'' The moderate use of hemp has, in fact, become a part of the domestic, social, and even religious life of the people, and it is accordingly considered by the government of the country unnecessary and inadvisable to attempt to prohibit it, even if that were possible, which is very doubtful.

As in the case of opium, occasional and exceptional harm from excess does not justify deprivation of what, in the vast majority of cases, is an innocuous luxury. At the same time, considering that there is a tendency to abuse and excessive consumption of all such agents, the government of India considers it right to place some restriction upon the sale of hemp drugs in the shape of "taxation to the highest point compatible with the prevention of illicit production in British India, and of smuggling from the native States.'' The government is also prepared to entrust municipal bodies with local option as regards the number of ganja shops, and to sanction the framing of rules prohibiting the grant of sale licenses to females, or the supply of the drugs to such sections of the community as are liable to abuse them.—*British Medical Journal.*

